# Identification of molecular patterns and diagnostic biomarkers in juvenile idiopathic arthritis based on the gene expression of m^6^A regulators

**DOI:** 10.3389/fped.2022.930119

**Published:** 2022-09-08

**Authors:** Shibo Zhang, Jing Qin, Yuechao Zhao, Jian Wang, Zhiliang Tian

**Affiliations:** Department of Pediatrics, The Second Affiliated Hospital of Harbin Medical University, Harbin, China

**Keywords:** m^6^A, juvenile idiopathic arthritis, immune microenvironment, long non-coding RNA, m^5^C, diagnostic model

## Abstract

The role of *N*^6^-methyladenosine modification in immunity is increasingly being appreciated. However, the landscape of m^6^A regulators in juvenile idiopathic arthritis (JIA) is poorly understood. Thus, this study explored the impact of m^6^A modification and related lncRNAs in JIA immune microenvironment. Fourteen m^6^A regulators and eight lncRNAs were identified as potential diagnostic biomarkers for JIA. Two diagnostic models for JIA were also constructed. The putative molecular regulatory mechanism of *FTO*-mediated m^6^A modification in JIA was hypothesized. Three distinct m^6^A patterns mediated by 26 m^6^A regulators and three diverse lncRNA clusters mediated by 405 lncRNAs were thoroughly investigated. They exhibited dramatically diverse immune microenvironments and expression of HLA genes. The identification of two separate subtypes of enthesitis-related arthritis implies that our work may aid in the establishment of a more precise categorization system for JIA. m^6^A modification-related genes were obtained, and their underlying biological functions were explored. The m^6^Ascore system developed for individual JIA patients may be utilized to evaluate the immunological state or molecular pattern, thereby offering therapy recommendations. In short, through the investigation of the m^6^A regulators in JIA, the current work may contribute to our knowledge of the pathophysiology of JIA.

## Introduction

Juvenile idiopathic arthritis (JIA) is defined as unexplained joint swelling and pain that lasts longer than 6 weeks in childhood (under 16 years of age) (1). JIA is the most frequent rheumatological chronic inflammatory illness in children, characterized by chronic synovitis and systemic multi-organ dysfunction. Neither the etiology nor the pathogenesis of JIA is clear. Furthermore, while JIA is a collective name for a set of disorders, their pathophysiology may differ. It is now believed that JIA is the consequence of environmental exposure in genetically susceptible individuals, and there is the involvement of infection, genetics, epigenetics, immunity, and other factors (2). Multiple hypotheses have been proposed to explain the pathophysiology of JIA. For instance, the specific components of various infectious microorganisms act as foreign antigens to genetically susceptible children, activating immune cells and inflicting damage and degeneration to self-tissues either directly or indirectly through the secretion of cytokines and autoantibodies that promote abnormal immune responses. Many studies have confirmed that JIA has a genetic basis, with human leukocyte antigens (HLA) being the most extensively examined. Individuals with the *HLA-DR4*, *HLA-DR5*, and *HLA-DR8* alleles are predisposed to JIA (3). JIA was formerly known as juvenile rheumatoid arthritis (JRA) by the American College of Rheumatology (ACR) and juvenile chronic arthritis (JCA) by the European Alliance of Associations for Rheumatology (EULAR). Not only were the names dissimilar, but they were also classified in different ways. It was not until 2001 that the International League Against Rheumatism (ILAR) defined a common classification standard for JIA (1). Although the ILAR categorization system has made a substantial contribution to JIA research, the limitations of this classification have been more evident in recent years (4). A more sophisticated categorization system for JIA based on pathogenesis and molecular biology is urgently required. Modern diagnostic methods for JIA rely heavily on clinical signs and the exclusion of other similar diseases. At this time, there are also no laboratory tests that can be used to diagnose JIA. As modern medicine advances by leaps and bounds, the prognosis of JIA has improved dramatically. However, certain children are afflicted with severe complications such as loss of joint function, lifelong eye damage owing to uveitis, and macrophage activation syndrome (MAS), which may be fatal (5, 6). Therefore, effective early diagnostic biomarkers or models of JIA are highly sought after.

Ribonucleic acid modification (RNA) is regarded as the third layer of epigenetics, and its role in biology is becoming increasingly appreciated (7). It has been shown that RNA has more than 100 distinct forms of post-synthesis modifications, including *N*^6^-methyladenosine (m^6^A), 5-methylcytosine (m^5^C), *N*^1^-methyladenosine (m^1^A), 3-methylcytosine (m^3^C), and *N*^1^-methylguanosine (m^1^G) (8). m^6^A is the most common post-transcriptional modification in eukaryotic cells, where it regulates the transcription, processing, splicing, degradation, and translation of RNA (9). m^6^A modifies and regulates RNA through dynamic interactions between the three homologous components, including methyltransferases (writers), binding proteins (readers), and demethylases (erasers) (10). m^6^A RNA methylation modification has been confirmed to play a pivotal role in a wide range of diseases, including cancers, immune-related illnesses, and neurodegenerative diseases (11–13). It has been demonstrated that m^6^A modification and m^6^A regulator proteins play prominent roles in regulating both innate and adaptive immunity (14). A study indicates that hnRNPA2B1 detects herpesvirus DNA, thereby increasing m^6^A modification to elicit innate immune responses (15). In mouse T cells, the deficiency of METTL3 impairs the differentiation and homeostasis of T cells (16). CD4^+^ regulatory T cells (Tregs) without m^6^A RNA modification were also reported to lose their systematic suppressive effect against immune cells (17). Additionally, m^6^A is crucial for the activation of dendritic cells (DCs), which produce co-stimulatory molecules and promote the activation of adaptive immune responses through antigen cross-presentation (14). Non-coding RNA is thought to be the foundation of epigenetic regulation (18). Long non-coding ribonucleic acids (lncRNAs) are characterized as transcripts with a length exceeding 200 nucleotides that do not undergo protein translation (19). lncRNAs have been proven to be pivotal regulators of mRNA processing, post-transcriptional regulation, and protein activity (20). There is a substantial correlation between m^6^A and lncRNAs. On the one hand, m^6^A modifications can affect the structure of the RNA-DNA triple helix, thereby regulating the association of lncRNAs with specific DNA loci; on the other hand, m^6^A modifications can also create binding sites for binding proteins or modify the structure of local RNAs, which in turn induce the binding of RNA-binding proteins (RBPs) to influence the function of lncRNAs (21, 22). Additionally, lncRNAs contain more m^6^A modification sites than mRNAs do (23). 5-methylcytosine (m^5^C) is another frequent post-transcriptional modification of RNA (24). m^5^C performs similar functions to m^6^A, and the methylation of m^5^C also requires the involvement of writers, erasers, and readers. However, it’s not clear how these modifications work in juvenile idiopathic arthritis.

This research was based on gene expression profiles of peripheral blood mononuclear cells (PBMCs) from JIA children and healthy controls. We systematically analyzed the molecular patterns mediated by m^6^A regulators and m^6^A-related lncRNAs, respectively. We undertook detailed assessments of the differences in biological function and immunological microenvironment across these patterns, as well as putative molecular explanations. Fourteen m^6^A regulators and eight lncRNAs were identified to be crucial for JIA diagnosis. They may serve as potential biomarkers for JIA diagnosis. Two diagnostic models were developed utilizing m^6^A regulators and lncRNAs, respectively. Furthermore, two models were tested using the same external dataset and then compared against one another to identify the superior model. Additionally, genes influencing the formation of distinct m^6^A patterns were identified, and their potential biological functions were elucidated. Two distinct patterns in enthesitis-related arthritis (ERA) were observed in our study, indicating that there may be two separate subtypes with distinct pathogenesis and progression in ERA. This also suggests that m^6^A regulators and lncRNAs may assist in establishing the novel JIA classification system. Finally, taking into account the individual heterogeneity of m^6^A modifications, the m^6^Ascore system was designed to estimate the m^6^A modification status of individual JIA patients.

## Materials and methods

### Microarray data collection

The analysis of this study was mainly based on five datasets from the GEO database^1^. All five datasets (GSE11083, GSE13501, GSE20307, GSE21521, and GSE67596) were produced using the same microarray platform of GPL570 (Affymetrix Human Genome U133 Plus 2.0 Array). This guaranteed the reliability of the merged analysis. Following the removal of duplicates, 196 JIA samples and 89 healthy samples were identified. Raw CEL files were extracted from the GEO supplementary files and assembled into a single dataset. The background adjustment and normalization were implemented using the rma function in the Bioconductor package affy (25). Non-biological batch effects among five datasets were eliminated using the ComBat algorithm (26). Probes without annotation information were removed. As for more than one probe corresponding to the same gene symbol, the median value was taken as the gene expression value. As an additional validation, we collected another independent dataset (GSE15645) to test the two diagnostic models we developed in this study.

### Different expression of m^6^A regulators between juvenile idiopathic arthritis and healthy controls

Pearson correlation analysis was executed in order to examine the co-expression patterns of 26 m^6^A RNA methylation regulators in all samples and just within JIA samples. Based on the Wilcox test, we systematically evaluated the different expressions of 26 m^6^A regulators between JIA samples and healthy control samples. All 26 m^6^A regulators were separately analyzed using univariate logistic regression to recognize JIA diagnosis-related m^6^A regulators (*p* < 0.05). The m^6^A regulators picked by univariate logistic regression were then submitted to LASSO (Least Absolute Shrinkage and Selection Operator) regression for dimensionality reduction analysis, and 10-fold cross-validation was applied. Following LASSO’s selection of m^6^A regulators, a diagnosis model for JIA was developed through multivariate logistic regression. Receiver operating characteristic (ROC) curves and the area under the curve (AUC) were used to estimate the prediction power of the JIA model.

### Three molecular patterns mediated by 26 m^6^A regulators

To observe the different m^6^A patterns in JIA samples, unsupervised hierarchical clustering analysis was employed. On the basis of the gene expression of 26 m^6^A regulators, we executed unsupervised consensus clustering with 1,000 replications using the ConsensusClusterPlus R package (27). According to published literature and the R package reference manual, the optimal number of clusters was calculated using the *K* value with the lowest proportion of ambiguously clustered pairs (PAC) (28). Gene set variation analysis (GSVA) was conducted to evaluate biological function and progress variations among these m^6^A patterns (29). The KEGG gene set (c2.cp.kegg.v7.5.1.symbols.gmt) from MSigDB was downloaded for running GSVA. Statistical significance was determined only for pathways with an adjusted *p*-value less than 0.05. Single-sample gene set enrichment analysis (ssGSEA) was used to assess the infiltration status of 23 immune cell types and immune reactions among m^6^A patterns. The gene signatures of each immune cell type were obtained from prior research (30). Seventeen immune reactions related gene sets were downloaded from the ImmPort database^2^ (31). The relationship between HLA and JIA has been extensively studied. Here we also examined the differences in HLA expression among m^6^A patterns. All tests were Wilcox unless otherwise noted.

### Identification of m^6^A modification-related genes

Genes that show significant differences in expression between different m^6^A patterns were identified using the limma R package (32). Specifically, genes with | Log_2_FC (fold-change)| > 1 and adjusted *p*-value < 0.05 were considered as DEGs. To gain a deeper insight into the biological functions of m^6^A modification-related genes, Gene Ontology (GO) and Kyoto Encyclopedia of Genes and Genomes (KEGG) enrichment analysis were conducted with the clusterProfiler R package (33). Using weighted gene correlation network analysis (WGCNA), we identified gene modules associated with different patterns to obtain the feature genes of each molecular pattern mediated by the m^6^A regulators (34).

### m^6^A- and m^5^C-related long non-coding ribonucleic acids

Pearson correlation analysis was used to assess the expression correlation between lncRNAs and m^6^A regulators. We defined m^6^A-related lncRNAs as those whose expression value was correlated with at least one of the 26 m^6^A regulators (| correlation coefficients| > 0.4 and *p* < 0.001). The same process was used to find m^5^C-related lncRNAs. Next, we considered lncRNAs that overlapped in both m^6^A-related lncRNAs and m^5^C-related lncRNAs as m^6^A- and m^5^C-related lncRNAs. Unsupervised clustering analysis was performed to explore the potential biological functions of m^6^A- and m^5^C-related lncRNAs in JIA. Using the same analysis process as described above, the degree of immune cells infiltration and immune reactions, as well as the HLA expression relationship were measured. The LASSO regression algorithm was then employed to screen important lncRNAs that could accurately discriminate JIA samples from normal samples. Using multivariate logistic regression, an additional diagnosis model was constructed based on lncRNA signatures. We compared the diagnostic capabilities of both models in order to select the model with the best performance.

### m^6^Ascore: Construction and application

Taking into consideration the individual phenotypic variation and intricacy of m^6^A modification, a scoring system was constructed to assess the m^6^A modification panorama of individual JIA patients based on the m^6^A modification-related genes. All samples were subjected to principal component analysis (PCA) of the m^6^A modification-related genes expression profiles. Summing the PC1 and PC2 scores of each sample yields the m^6^Ascore for the sample. m^6^Ascore was compared between the different patterns. JIA children were divided into two groups based on low or high m^6^Ascore. Further assessment was made of the differences in immune microenvironment between patients with low and high m^6^Ascore.

## Results

### The landscape of m^6^A regulators in juvenile idiopathic arthritis

**FIGURE 1:**
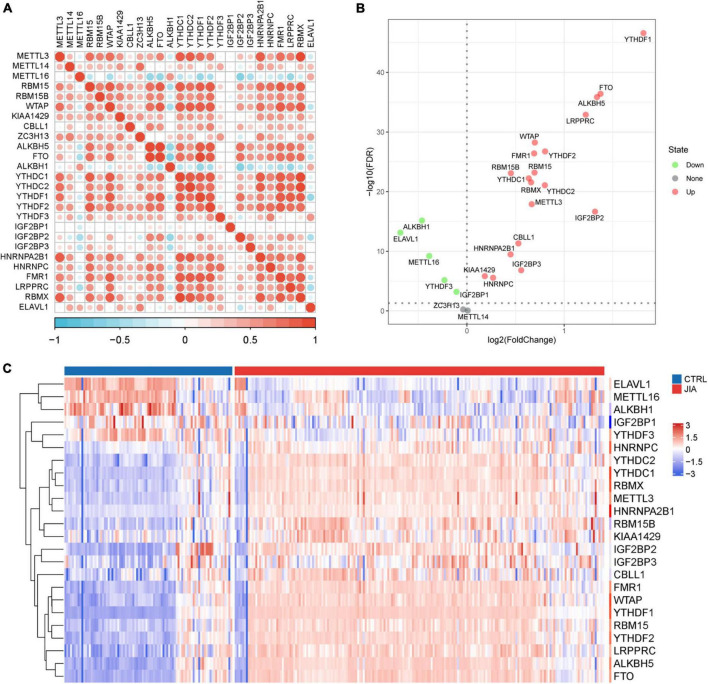
The landscape of m^6^A regulators in juvenile idiopathic arthritis (JIA). **(A)** The co-expression network of 26 m^6^A regulators. The upper triangle is the network in all samples, and the lower triangle is the network in just JIA samples. **(B)** The volcano plot shows the differential expression of m^6^A regulators between JIA and healthy controls samples. **(C)** The heatmap plot of 24 significantly differentially expressed m^6^A regulators.

**FIGURE 2:**
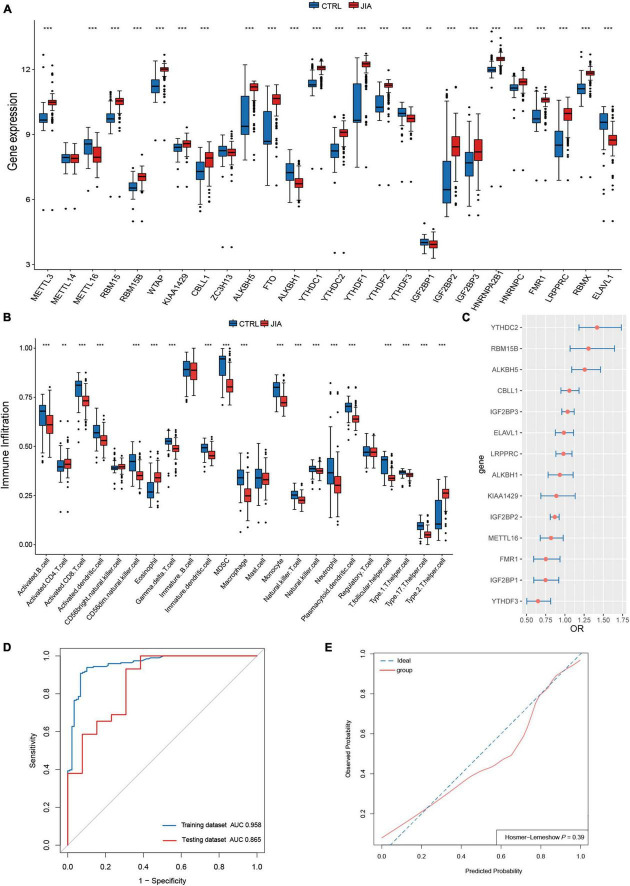
A diagnostic model was constructed with m^6^A regulators for juvenile idiopathic arthritis (JIA). **(A)** Comparison of the expression of 26 m^6^A regulators in JIA and normal control samples. **(B)** The discrepancies in the abundance of each infiltrating immunocyte between JIA and healthy samples. **(C)** Relative risks of 14 diagnostic m^6^A regulators for JIA were calculated. **(D)** The developed diagnostic model for JIA was evaluated by the ROC curve and AUC value. **(E)** Hosmer–Lemeshow (H–L) test was used to evaluate the fit of the developed model.

In a review of published studies, 26 m^6^A regulators were identified, including nine writers, three erasers, and fourteen readers (Supplementary Table 1). The co-expression network between m^6^A regulators was first investigated (Figure 1A). There was a broad and notable correlation observed among the expression of m^6^A regulators. In all samples, there were two erasers (*FTO* and *ALKBH5*) that were the most relevant m^6^A regulators in expression, while in only JIA samples, they were two readers (*YTHDC1* and *RBMX*). These results suggest they could function synergistically during the course of JIA. *YTHDF1* was the m^6^A regulator with the biggest fold change and the most pronounced statistical significance in JIA samples compared to healthy samples, suggesting that it may play an indispensable role in JIA pathogenesis (Figure 1B). Each type of m^6^A regulator had a unique regulator that behaved differently from the others. Other writers exhibited a positive relationship with erasers and readers, as opposed to *METTL16*, which had a broad negative relationship. Similarly, *ALKBH1* did not show a positive correlation with other m^6^A regulators as did other erasers, but rather a negative correlation. The same goes for *ELAVL1*. The results above indicated that m^6^A regulators mediate an intricate network of RNA methylation modifications in JIA. There were prominent differences in m^6^A regulators expression between JIA and healthy samples (except for *METTL14* and *ZC3H13*), and JIA generally had higher grades of m^6^A regulators expression (Figures 1C, 2A). Significant differences were observed between JIA and healthy samples in the immune cell infiltration (Figure 2B). Notably, the ratio of CD4 T cell to CD8 T cell was dramatically higher in JIA patients than in healthy controls, indicating that JIA was an autoimmune disease. However, no statistically significant change in the regulatory T (T_reg_) cell was observed, suggesting that the T_reg_ cell may not be implicated in the etiology of JIA. This finding has been highlighted in previous research and was further supported here (35). Notably, type 2 T helper cell (Th2) was active in JIA, and their release of numerous cytokines could directly affect the inflammatory process in JIA. We hypothesized that the high level of eosinophils in JIA released a large amount of interleukin 4 (IL-4), which could induce the differentiation of Th0 cells to Th2 cells.

### A diagnostic model constructed with m^6^A regulators for juvenile idiopathic arthritis

Given the noteworthy expression difference of m^6^A regulators between JIA and normal samples, we considered m^6^A regulators for JIA diagnosis. The construction of the diagnostic model also provided us with a better knowledge of the role of m^6^A regulators in JIA. The first step was to identify 24 m^6^A regulators associated with JIA diagnosis through univariate logistic regression analysis. It turns out that these 24 m^6^A regulators are the same as the differentially expressed m^6^A regulators from the previous step. LASSO regression analysis with 10-fold cross-validation was employed to filter out m^6^A regulators that were relatively insignificant to the diagnosis of JIA. Finally, fourteen m^6^A regulators (*METTL16*, *RBM15B*, *KIAA1429*, *CBLL1*, *ALKBH5*, *ALKBH1*, *YTHDC2*, *YTHDF3*, *IGF2BP1*, *IGF2BP2*, *IGF2BP3*, *FMR1*, *LRPPRC*, and *ELAVL1*) crucial to diagnosing JIA were obtained. Multivariate logistic regression analysis was performed to construct the diagnostic model of JIA with these 14 m^6^A regulators as variables. In addition, the odds ratio (OR) with a 95% confidence interval (CI) of each variable was calculated (Figure 2C). By tracing the ROC curve of the model, we could see that it was well suited to distinguish JIA from healthy samples (Figures 2D,E). The concordance index (C-index) of this model was 0.96 (95% CI 0.93–0.98), and the area under the curve (AUC) was 0.958 (95% CI 0.933–0.982). The diagnostic model was validated using the independent dataset GSE15645 to evaluate its reliability against an external independent dataset. The AUC value of the validation dataset was 0.865 (95% CI 0.739–0.991). The external validation result indicated that the derived model had a fair prediction capacity against an external dataset.

### Identification of distinct m^6^A patterns in juvenile idiopathic arthritis

**FIGURE 3:**
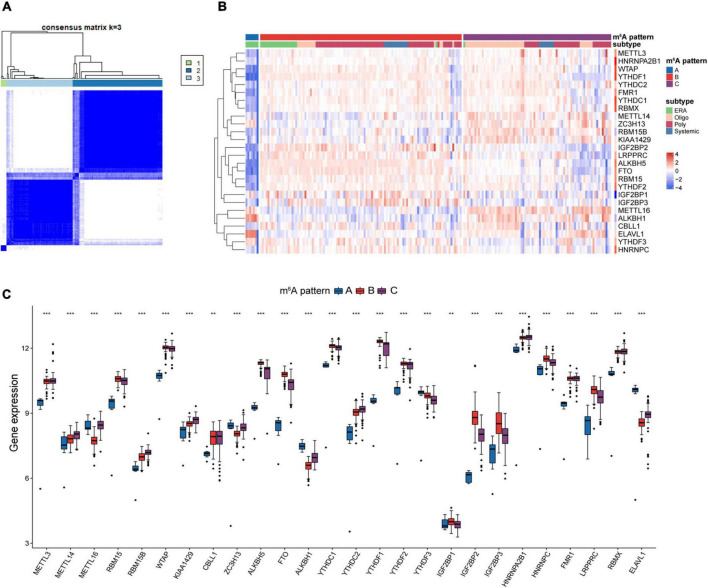
Unsupervised clustering analysis of 26 m^6^A regulators. **(A)** Consensus clustering classified juvenile idiopathic arthritis (JIA) into three m^6^A patterns. **(B)** Differences between the m^6^A patterns and extant classification system. **(C)** Different expressions of the m^6^A regulators in the three m^6^A patterns.

Unsupervised clustering analysis of JIA samples based on the 26 m^6^A regulators transcripts was used to examine the multiple m^6^A patterns in JIA. A total of three distinct m^6^A patterns were identified, with seven samples falling into pattern A, 109 samples falling into pattern B, and 80 samples falling into pattern C (Figure 3A). The three m^6^A patterns discovered were inconsistent with the current clinical categorization system that focused primarily on patient symptoms (Figure 3B). Enthesitis-related arthritis (ERA) samples were observed to be split into two m^6^A patterns: 7 cases in pattern A and 22 cases in pattern B, indicating that two different molecular environments may characterize ERA. Each of the 26 m^6^A regulators showed considerable variation across these three m^6^A patterns, demonstrating the presence of various molecular patterns in JIA (Figure 3C). Pattern A generally showed lower expression of m^6^A regulators than pattern B or C, with a handful of exceptions (*ZC3H13*, *ALKBH1*, *YTHDF3*, *ELAVL1*) showing strongly high-expressed.

### Divergent immune microenvironments in juvenile idiopathic arthritis

**FIGURE 4:**
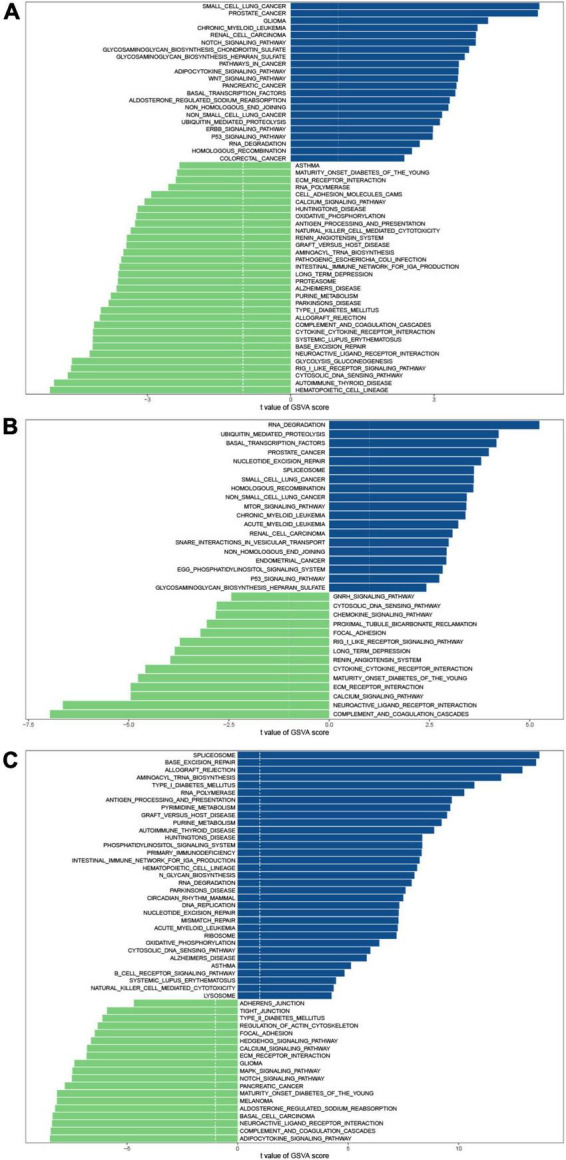
GSVA enrichment analysis shows the differences in the underlying biological processes between the three m^6^A patterns. **(A)** Pattern B vs. Pattern A. **(B)** Pattern C vs. Pattern A. **(C)** Pattern C vs. Pattern B.

To investigate the biological differences among these three m^6^A patterns, we employed GSVA enrichment analysis to explore the KEGG pathways associated with each pattern. Compared with patterns B and C, pattern A was mainly enriched in the activation of immune related-pathways such as antigen processing and presentation, natural killer cell-mediated cytotoxicity, the intestinal immune network for IgA production, cytokine-cytokine receptor interaction, chemokine signaling pathway, and RIG I like receptor signaling pathway (Figures 4A,B). Additionally, matrix-related pathways such as ECM-receptor interaction and cell adhesion molecules (CAMs) could be discovered in pattern A. Pattern B revealed a slew of signaling pathways, including Notch signaling pathway, adipocytokine signaling pathway, Wnt signaling pathway, ErbB signaling pathway, p53 signaling pathway, Calcium signaling pathway, and MAPK signaling pathway (Figures 4A,C). It was also discovered that pattern A was linked to focal adhesion, and pattern B was connected with the regulation of actin cytoskeleton. Focal adhesion is the physical link between the cell actin cytoskeleton and extracellular matrix (ECM). Macrophage activation syndrome (MAS) is the most dangerous complication of JIA. Genome-Wide Association Studies (GWAS) investigations reveal that the aberrations in cellular cytotoxicity as the key disfunction in MAS may be caused by derangements in cellular assembly and cytoskeletal organization. Therefore, we speculated that additional research into the cytoskeleton would aid in our understanding of the molecular processes driving JIA development. In comparison to pattern B, pattern C also exhibited immune-related pathways enrichment. Ubiquitin-mediated proteolysis was more prevalent in pattern C than in pattern A, indicating that ubiquitination modifications may have a place in pattern C. A number of autoimmune disorders, malignancies, and neurodegenerative diseases were also identified in the enrichment analysis, suggesting that m^6^A modification may also participate in their pathogenesis. Many published studies have proved the association (36–38).

**FIGURE 5:**
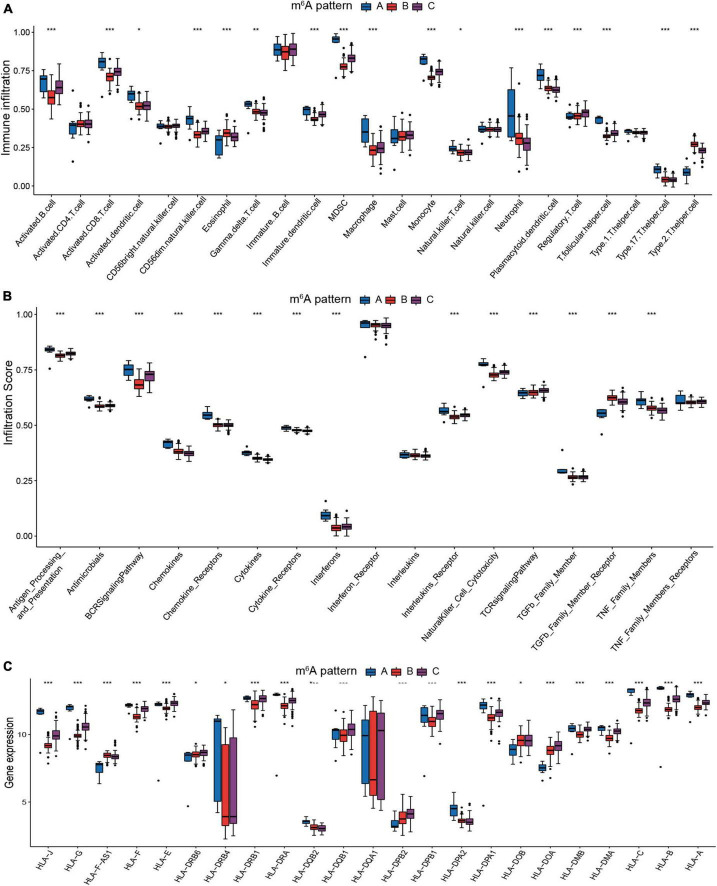
The immunological microenvironment varies significantly across the three m^6^A patterns. **(A)** The discrepancies in the abundance of each infiltrating immunocyte in the three m^6^A patterns. **(B)** The three patterns exhibit varying degrees of immunological response. **(C)** Human leukocyte antigens (HLA) gene expression varied significantly across the three m^6^A patterns.

Due to the substantial association between the three patterns and immunity, we analyzed their variations in immune cells infiltrating, immune response processes, and HLA genes expression to recognize the immune microenvironment landscapes of three m^6^A patterns. In comparison to patterns B and C, pattern A exhibited a higher overall infiltration of immune cells (Figure 5A). Autoantibodies are often detected in JIA patients, and many studies have sought to determine the role of autoantibodies in JIA. Despite the fact that autoantibodies for JIA have not been found to be diagnostic, they have been proven to assist in assessing the severity of the condition. The Childhood Arthritis and Rheumatology Research Alliance (CARRA) has identified rheumatoid factor (RF) as a risk factor for poor prognosis in individuals with polyarticular JIA (39). Given the greater infiltration of activated B cells in pattern A compared to the other two patterns, it is hypothesized that autoantibodies were more likely to be detected in pattern A. Subsequent immune response analysis demonstrated pattern A exhibited more robust immunological responses, particularly in the humoral immunity (Figure 5B). Pattern A exhibited more activity in antigen processing and presentation, BCR signaling pathway, chemokines, chemokines receptors, cytokines, cytokine receptors, and natural killer cell cytotoxicity. Pattern B only exceeded the other patterns at the level of eosinophils and Th2 cells infiltration. On the other hand, Pattern A had the lowest amounts of eosinophils and Th2 cells infiltration. The co-expression of eosinophils and Th2 may validate our previous hypothesis that eosinophils regulated Th2 expression through releasing IL4. The infiltration degree of each immune cell in pattern C was mostly moderate. As for the immune reaction, pattern C also exhibited a moderate degree, whereas pattern B exhibited the least. There was no significant difference in activated CD4 T cell infiltration across three patterns, indicating that activated CD4 T cell was irreplaceable in all three m^6^A patterns. Similar trends can be detected in the expression of the HLA genes. The expression of HLA was generally higher in pattern A than in the other two patterns (Figure 5C). HLA gene expression varied significantly between the three m^6^A patterns, hinting this characteristic could serve as a criterion for the new categorization of JIA.

### *FTO*-mediated regulations of immune microenvironment in juvenile idiopathic arthritis

**FIGURE 6:**
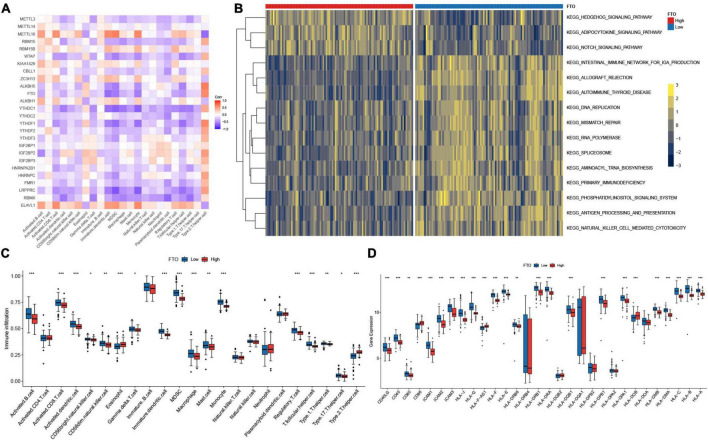
*FTO*-mediated regulations of the immune microenvironment in juvenile idiopathic arthritis (JIA). **(A)** Correlations between m^6^A regulators and the immunocytes. **(B)** GSVA enrichment analysis shows the differences in the underlying biological processes between the JIA patients with high and low expression of *FTO*. **(C)** The discrepancies in the abundance of each infiltrating immunocyte between the JIA patients with high and low expression of *FTO.* **(D)** Patients with low-expressed *FTO* exhibited significantly higher expression of co-stimulatory molecules, adhesion molecules, and MHC.

In addition, we checked for the correlation between each immune cell type and each m^6^A regulator using Spearman correlation analysis (Figure 6A). Here, we concentrated on *FTO*, which showed a considerable negative connection with various immune cells. FTO was the first m^6^A demethylase to be identified (40). The identification of FTO demonstrated that m^6^A modification is dynamic and reversible. To assess immunological disparities between JIA patients with high and low expression of *FTO*, JIA patients were split into two groups based on the expression level of *FTO*, using the mean value as a threshold. GSVA enrichment pathways demonstrated that patients with reduced *FTO* expression were associated with immunological activation and m^6^A-related regulatory mechanisms, such as antigen processing and presentation, natural killer cell mediated cytotoxicity, IgA production, DNA replication, mismatch repair, spliceosome, and aminoacyl-tRNA biosynthesis (Figure 6B). Further comparative analysis of immune cell infiltration in two groups revealed a significant increase in patients with low expression of *FTO* (Figure 6C). Notably, highly expressed activated dendritic cells were observed in patients with low expression of *FTO*. Activation of dendritic cells (DCs) requires the involvement of major histocompatibility complex (MHC), co-stimulatory molecules, cytokines, and chemokine receptors (41). Then we investigated the interaction between *FTO* and these molecules to learn how the expression of *FTO* affected DCs activation. The result showed that patients with low-expressed *FTO* exhibited significantly higher expression of co-stimulatory molecules, adhesion molecules, and MHC (Figure 6D). From the above results, we hypothesized that *FTO*-mediated m^6^A methylation modification might stimulate the production of MHC and co-stimulatory molecules and consequently activate conventional dendritic cells, enhancing the adaptive immune reaction in JIA patients.

### m^6^A modification-related genes and weighted gene correlation network analysis

**FIGURE 7:**
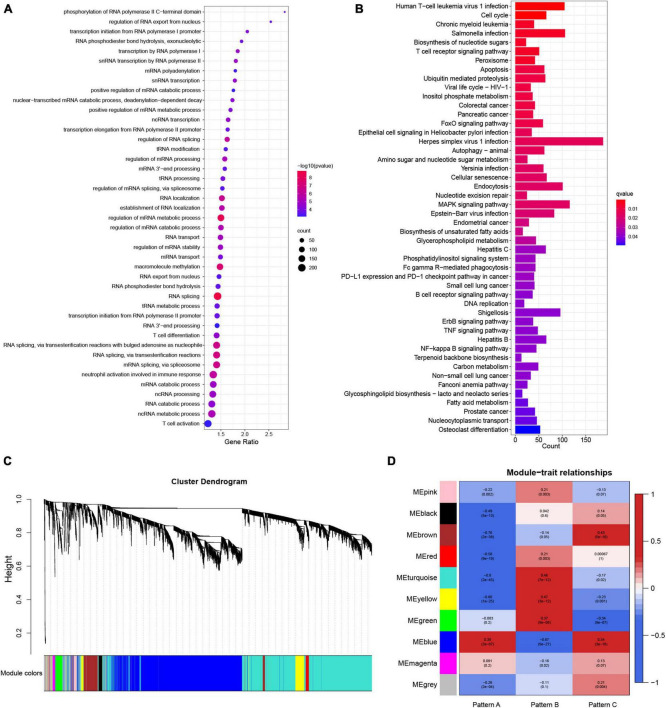
The underlying biological processes of m^6^A modification-related genes and weighted gene correlation network analysis (WGCNA) of the top 25% variation genes. **(A)** Partial results of Gene Ontology (GO) enrichment analysis for m^6^A modification-related genes in terms of biological processes (BP). **(B)** The Kyoto Encyclopedia of Genes and Genomes (KEGG) pathway analysis of m^6^A modification-related genes. **(C)** Identified modules by WGCNA. **(D)** Relationships between modules and traits.

To gain further insight into the mechanism underlying the formation of different molecular patterns, differentially expressed genes among the three m^6^A patterns were identified. The union of three DEGs sets was defined as the m^6^A modification-related genes. A total of 6919 m^6^A modification-related genes were obtained. The biological processes of GO enrichment analysis demonstrated that the genes were remarkably enriched in m^6^A-related regulatory mechanisms and immunity (Figure 7A). Similar results were noted in the KEGG pathway analysis (Figure 7B). Gene modules linked with diverse molecular patterns were identified using WGCNA. In total, nine gene modules were obtained (Figure 7C). For each m^6^A pattern, there were several modules substantially associated with it (Figure 7D). These gene modules may shed light on the m^6^A modification patterns from a more refined genetic perspective.

### m^6^A- and m^5^C-related long non-coding ribonucleic acids

**FIGURE 8:**
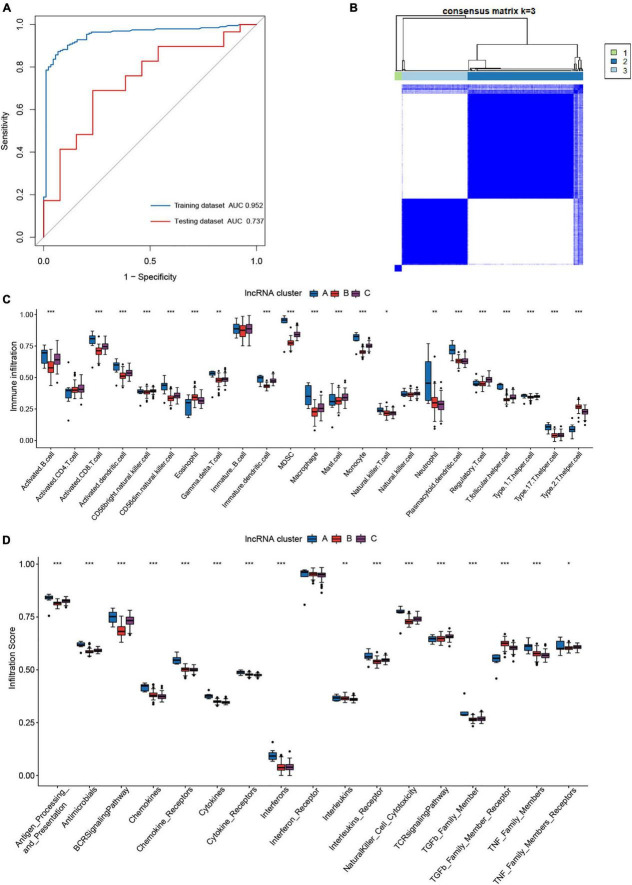
The role of m^6^A- and m^5^C-related long non-coding ribonucleic acids (lncRNAs) in juvenile idiopathic arthritis (JIA). **(A)** The ROC curve of the second diagnostic model constructed with lncRNAs for JIA. **(B)** Consensus clustering classified JIA into three lncRNA clusters. **(C)** The discrepancies in the abundance of each infiltrating immunocyte in the three lncRNA clusters. **(D)** The three clusters exhibit varying degrees of immunological response.

Our attention has been drawn to the fact that lncRNAs have more m^6^A modification sites than mRNAs (23). m^5^C modification is another frequently occurring RNA methylation modification in humans. To get a better understanding of how m^6^A regulators regulate the immunological microenvironment in JIA and the intricate lncRNAs regulatory network generated by m^6^A and m^5^C regulators, the m^6^A- and m^5^C-related lncRNAs were identified in this study. On the basis of published data, 14 m^5^C regulators were identified, including ten writers, three erasers, and one reader (Supplementary Table 2). The Pearson correlation analysis revealed 500 m^6^A-related lncRNAs and 442 m^5^C-related lncRNAs. Eventually, a total of 405 m^6^A- and m^5^C-related lncRNAs were identified. Given the excellent performance of the diagnostic model for JIA conducted using the above 14 m^6^A regulators, we investigated if m^6^A- and m^5^C-related lncRNAs were also capable of diagnosing JIA. LASSO regression analysis with 10-fold cross-validation was performed to identify lncRNAs essential for JIA diagnosis. Finally, we collected eight lncRNAs (*ZNF582-AS1*, *ZNF503-AS2*, *UBL7-AS1*, *TTTY5*, *PRKAG2-AS1*, *LINC01007*, *LINC00424*, and *ADARB2-AS1*) for use in the development of the diagnostic model. The diagnostic model for JIA, which was based on the expression of eight lncRNAs, was developed using the same multivariate logistic regression methodology as previously described. The calculated AUC was 0.952 (95% CI 0.925–0.978). The model was further tested against the independent dataset GSE15645, and the AUC was 0.737 (95% CI 0.572–0.903) (Figure 8A). The comparison of AUC values indicated that the diagnostic model developed using m^6^A regulators was superior to the model constructed using lncRNAs.

**FIGURE 9:**
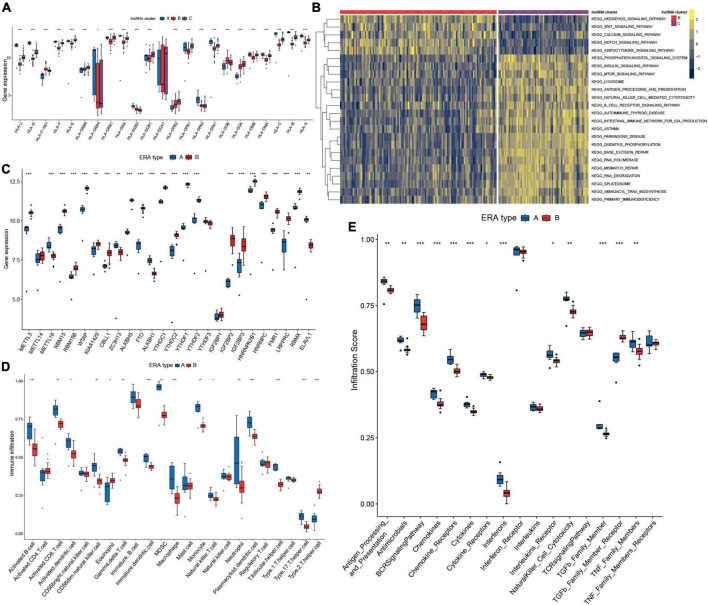
Two distinct subclusters of enthesitis-related arthritis (ERA) were identified. **(A)** Human leukocyte antigens (HLA) gene expression varied significantly across the three long non-coding ribonucleic acid (lncRNA) clusters. **(B)** GSVA enrichment analysis shows the differences in the underlying biological processes between the three lncRNA clusters. **(C)** Different expressions of the m^6^A regulators in the two subclusters of ERA. **(D)** The discrepancies in the abundance of each infiltrating immunocyte in the two subclusters of ERA. **(E)** The two subclusters of ERA exhibit varying degrees of immunological response.

The ConsensusClusterPlus R package was used again to classify JIA patients into three unique molecular patterns based on the expression of m^6^A- and m^5^C-related lncRNAs, including 7 cases in pattern A, 120 cases in pattern B, and 69 cases in pattern C (Figure 8B). To differentiate them, we referred to these three patterns as lncRNA cluster A, lncRNA cluster B, and lncRNA cluster C. To our astonishment, samples classified as lncRNA cluster A were identical to those classified as pattern A for m^6^A typing. A synergistic relationship between m^6^A regulators and lncRNAs may be shown here. The same immune infiltration analysis was performed for the three lncRNA clusters, and the results revealed a more subtle regulatory network. The three lncRNA clusters demonstrated substantial differences in the activated dendritic cell, CD56 bright natural killer cell, mast cell, and Type 1 T helper cell that were not found in the m^6^A patterns (Figure 8C). Moreover, in other immune cell types, both the lncRNA clusters and m^6^A patterns produced almost identical outcomes. The distinctions in immune reactions between the three lncRNA clusters were subsequently investigated. As expected, the lncRNA clusters were likewise more distinct than the m^6^A patterns (Figure 8D). Not only were the differential immune responses between m^6^A patterns present in lncRNA clusters, but also there were apparent discrepancies of interleukins and TNF family members receptors between lncRNA clusters. The analysis of HLA gene expression in lncRNA clusters revealed similar results to m^6^A patterns (Figure 9A). The above results demonstrated the inextricable link between m^6^A-mediated methylation modification and lncRNAs, and the collaborative participation of m^6^A and lncRNAs in the epigenetic modification of JIA. Since we addressed pattern A in detail previously, here we concentrate on the variations of biological pathways between lncRNA cluster B and C. LncRNA cluster C demonstrated greater immunological activity than lncRNA cluster B (Figure 9B).

### Two distinct subclusters of enthesitis-related arthritis

Pattern A in m^6^A typing and lncRNA cluster A in lncRNA typing were determined to be the same and match the ERA subtype of JIA, whereas the remaining ERA samples were classified into pattern B. ERA piqued our interest because of the two unique types presented. We defined them as type A and type B. The expression of 26 m^6^A regulators in the two types was investigated and compared (Figure 9C). The majority of m^6^A regulators were discovered to express at substantially lower levels in type A than in type B, including the *FTO* previously addressed in detail. Immune cells infiltrated at much higher quantities in type A (Figure 9D). Both humoral and cellular immunity were greater in type A than in type B. Further immune response analyses revealed that type A elicited a more robust immune response (Figure 9E). The two distinct ERA types indicated that the existing classification method of JIA was unable to differentiate individuals with different molecular subtypes, suggesting that m^6^A typing may aid in the discovery of novel classification approaches for JIA.

### Correlation between m^6^Ascore and immune microenvironment

**FIGURE 10:**
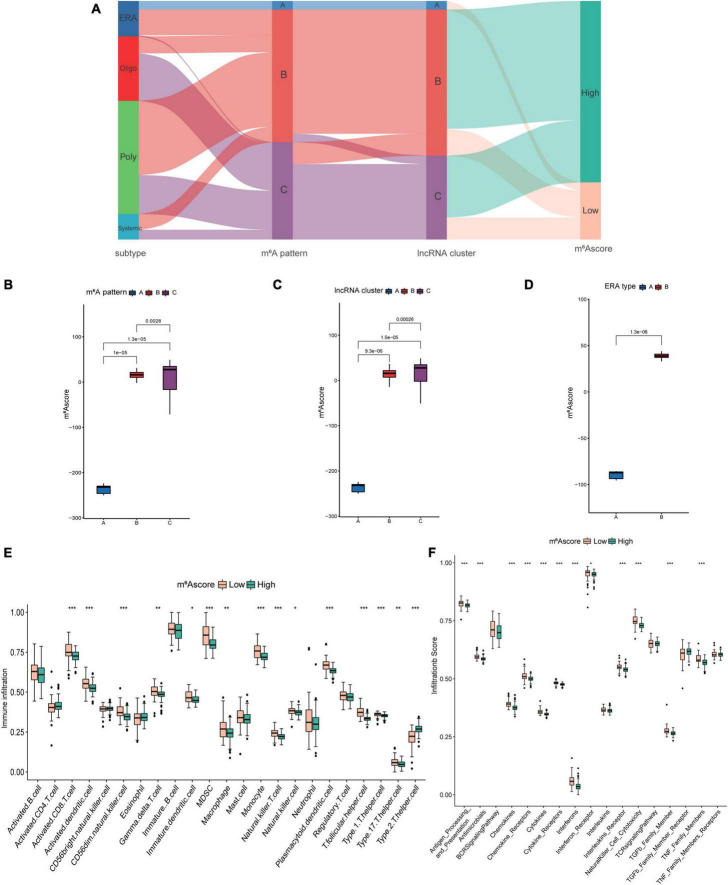
The construction and application of m^6^Ascore. **(A)** Alluvial diagram showing the changes of juvenile idiopathic arthritis (JIA) subtypes, m^6^A patterns, long non-coding ribonucleic acids (lncRNA) clusters, and m^6^Ascore. **(B)** Differences in m^6^Ascore between the three m^6^A patterns. **(C)** Differences in m^6^Ascore between the three lncRNA clusters. **(D)** Differences in m^6^Ascore between the two subclusters of enthesitis-related arthritis (ERA). **(E)** The discrepancies in the abundance of each infiltrating immunocyte between the high and low m^6^Ascore groups. **(F)** The high and low m^6^Ascore groups exhibit varying degrees of immunological response.

The preceding research conducted on patient groups was insufficient to predict the m^6^A methylation modification of individual JIA patients correctly. A scoring system based on the m^6^A modification gene signatures was developed to quantify the m^6^A modification in individual JIA patients. The Sankey diagram illustrated the changes of the JIA subtypes, m^6^A patterns, lncRNA clusters, and m^6^Ascore (Figure 10A). Wilcox test revealed statistical differences in m^6^Ascore between three m^6^A patterns (Figure 10B). Pattern A had the lowest m^6^Ascore, which means that lower m^6^Ascore may be linked to better immune reactions. Significantly different m^6^Ascores were also observed in the three lncRNA clusters and two ERA types (Figures 10C,D). The analysis of the overall immune cell infiltration landscape revealed that the low m^6^Ascore group tended to have an upregulated immune infiltration level (Figure 10E). It was also discovered that patients with low m^6^Ascore had higher levels of immune response-related biological processes (Figure 10F). These results suggest that the m^6^Ascore may be utilized to evaluate the immunological state of JIA patients, thereby offering therapy recommendations.

## Discussion

The limitations of the present JIA categorization system are becoming more obvious, necessitating the development of a novel classification system for JIA (4). Regardless of the distinctions across JIA subtypes, their systematic study allows the identification of pathogenic pathways by focusing on the functional networks rather than individuals. JIA is a rheumatological chronic inflammatory disease, so the importance of immunity for JIA is hard to overstate. m^6^A modification and lncRNA have been proven to have critical regulatory roles in immunity (14, 42, 43). The investigation was carried out utilizing the gene expression profiles of PBMCs from 196 JIA and 89 healthy control samples. First, we compared the gene expression of m^6^A regulators in JIA with healthy control samples. Of the 26 m^6^A regulators investigated, 24 exhibited statistically significant changes. It suggested that the m^6^A modification may play a vital role in the pathogenesis of JIA. Most of the m^6^A regulators showed higher levels of expression among the JIA individuals. This phenomenon has also been observed in specific tumor types, including lung cancer, gastric cancer, and breast cancer (10). However, the precise molecular mechanisms of this regulation remain unknown. This also suggests the need for more precise and direct methods to assess the m^6^A modification landscape in JIA, thus revealing the complex crosstalk of m^6^A regulators. The LASSO regression analysis screened out 14 m^6^A regulators critical for JIA diagnosis, and they served as the foundation for the development of the first logistic diagnostic model. After 405 m^6^A- and m^5^C-related lncRNAs were obtained, the LASSO regression analysis algorithm was reapplied to determine eight diagnostically significant lncRNAs (*ZNF582-AS1*, *ZNF503-AS2*, *UBL7-AS1*, *TTTY5*, *PRKAG2-AS1*, *LINC01007*, *LINC00424*, and *ADARB2-AS1*). Of these eight lncRNAs, *ZNF582-AS1* has been proved to regulate the m^6^A modification of *MT-RNR1*, hence facilitating the development and metastasis of renal clear cell carcinoma (44). One study discovered that *UBL7-AS1* promotes the proliferation of glioblastoma cells (45). And *PRKAG2-AS1* has been identified as a prognostic gene signature of esophageal squamous cell cancer (46). These eight lncRNAs may also play critical roles in JIA. The second diagnostic model for JIA was created in the same manner using these lncRNAs. ROC curves demonstrated that both models had a high diagnostic capacity. They were validated on an independent external dataset to compare their performance. It was discovered that the model developed by m^6^A regulators outperformed the other. These fourteen m^6^A regulators and eight lncRNAs may be potential biological diagnostic indicators or therapeutic targets of JIA. They may give guidance for future studies on JIA in general.

*FTO* and *ALKBH5* were the most significantly co-expressed m^6^A regulators in all samples, and their expression levels were inversely linked with the quantity of immune infiltrating cells. The role of *FTO* in JIA was thoroughly investigated, and potential molecular mechanisms of *FTO* were explored. *FTO* is also known as the adiposity-related gene (47). FTO was found as the first m^6^A demethylase, demonstrating that m^6^A modification is dynamic and reversible (40). *FTO* has been reported to modulate targets genes expression by decreasing the level of m^6^A in transcripts, thereby promoting hematopoietic cell transformation and leukemogenesis (48). It also has been shown that over-expression of *FTO* promotes the development of esophageal cancer through modulating lncRNA (49). In this study, we found that the expression of co-stimulatory molecules, adhesion molecules, and MHC were enhanced across the board in patients with decreased FTO expression. Additionally, patients with low-expressed *FTO* also had increased infiltration of conventional DCs and a more robust immunological response. Therefore, we postulated that *FTO*-mediated m^6^A modification may increase MHC and co-stimulatory molecules production, thereby activating conventional dendritic cells and enhancing immunological response in JIA patients. However, this theory must be validated by further trials.

Unsupervised clustering of JIA samples revealed three distinct m^6^A patterns. Further analysis demonstrated that pattern A had a much higher level of immune cells infiltration and a more vigorous immunological response than the other two patterns. m^6^A modification-related genes were obtained, and their putative biological functions were explored. m^6^A modification-related regulatory mechanisms and activation of immunity were significantly over-represented among the genes. Thus, they helped us comprehend the pathophysiology of JIA from the viewpoint of m^6^A modification. WGCNA analysis revealed gene modules associated with diverse m^6^A patterns, assisting in the comprehension of the m^6^A regulator-mediated molecular functional network from a genetic perspective. Similarly, another unsupervised clustering analysis on JIA samples was performed based on 405 m^6^A- and m^5^C-related lncRNAs, and three distinct clusters were identified, with cluster A exhibiting the highest level of immunity. Additionally, we discovered that molecular subtypes mediated by lncRNAs were more sophisticated than those mediated by m^6^A regulators since there were more significant variations in immune cells infiltration, immunological response, and HLA genes expression amongst lncRNA clusters than m^6^A patterns. Our investigation of GSVA enrichment analysis among different modification patterns revealed a large number of diseases such as asthma, leukemia, Huntington’s disease, Alzheimer’s disease, Parkinson’s disease, autoimmune thyroid disease, and a variety of cancers. Research on the link between m^6^A modification and cancer has been exhaustive (10). Our findings may imply that m^6^A modification also plays a crucial role in these diseases, pointing the way forward for further research into these disorders. It has been established that m^6^A is associated with several disorders as mentioned above (38, 50). JIA has been shown to have substantial and well-documented relationships with HLA alleles (51). Significant changes in HLA genes expression across distinct m^6^A patterns were observed in this study, suggesting that HLA genes may contribute to JIA categorization and aid in establishing a novel JIA classification system. Additionally, the two unique patterns seen in ERA subtypes indicated that the existing JIA categorization scheme was insufficiently detailed. These findings may help us comprehend the varied immunological microenvironments in JIA, as well as the diverse molecular biological processes that may exist in JIA, and may lead to the development of more effective and tailored treatment options for JIA patients.

The discrepancies in the gene expression of diverse m^6^A patterns were evidenced to be strongly connected with m^6^A modification and immune-related molecular mechanisms in this research. A scoring system to assess the m^6^A modifications in individual JIA patients was developed due to the heterogeneity of JIA. Both m^6^A regulators typing and lncRNAs typing revealed considerable differences in m^6^Ascore among subtypes. And m^6^Ascore displayed a substantial and inverse association with the immunological response. The management of patients may be greatly aided by the identification of certain molecular subtype or immunological status. The establishment of m^6^Ascore enables the development of personalized treatment strategies for JIA patients.

Our study is the first to conduct a comprehensive analysis of the m^6^A regulators mediated molecular patterns in juvenile idiopathic arthritis. We have shed light on the potential molecular pathogenesis of JIA from an immunological perspective. We did not limit our investigation to m^6^A modification of mRNA, and we also explored the impact of m^6^A- and m^5^C-related lncRNAs in JIA. This study demonstrated that m^6^A modification is involved in the regulation of the immunological microenvironment of JIA and plays a crucial role in its progression. However, we must acknowledge that this research has certain flaws. Due to the absence of clinical data, the relationship between m^6^A patterns and clinical features was not extensively investigated in this research. Currently, clinical diagnosis of JIA for physicians primarily relies on differential diagnosis to rule out other similar disorders. But it is unknown if the models we developed can distinguish JIA from other similar diseases. Besides, this study relies on bioinformatics analysis. Although the conclusions are theoretically valid, they have not been empirically tested and must be confirmed by further research. Nevertheless, we hope this research could shed fresh light on establishing the new JIA classification criteria and the future direction of JIA research.

## Conclusion

In conclusion, our study elucidates putative regulatory mechanisms behind the m^6^A modification in the immunological microenvironment of juvenile idiopathic arthritis. The analysis of m^6^A patterns and m^6^A- and m^5^C-related lncRNAs enables us to better understand the epigenetic landscape in JIA. The comprehensive assessment of m^6^A regulators in JIA will aid in the development of more effective and tailored immunotherapy for JIA patients.

## Data availability statement

The datasets analyzed for this study can be found in the Gene Expression Omnibus (https://www.ncbi.nlm.nih.gov/geo/query/acc.cgi?acc=GSE11083/GSE13501/GSE20307/GSE21521/GSE67596/GSE15645).

## Author contributions

ZT conceived and supervised the study. ZT and SZ designed the work. SZ, JQ, and YZ contributed to the data analysis. SZ, JQ, and JW wrote the manuscript. All authors contributed to the discussion and revision of the final manuscript.

## Footnotes

1
https://www.ncbi.nlm.nih.gov/geo

2
https://www.immport.org
